# Global Mapping of Interventions to Improve Quality of Life of Patients with Cancer: A Protocol for Literature Mining and Meta-Analysis

**DOI:** 10.3390/ijerph192316155

**Published:** 2022-12-02

**Authors:** Long Bao Nguyen, Linh Gia Vu, Xuan Thanh Nguyen, Anh Linh Do, Cuong Tat Nguyen, Laurent Boyer, Pascal Auquier, Guillaume Fond, Carl A. Latkin, Roger C. M. Ho, Cyrus S. H. Ho

**Affiliations:** 1 Institute for Preventive Medicine and Public Health, Hanoi Medical University, Hanoi 100000, Vietnam; 2 Institute for Global Health Innovations, Duy Tan University, Da Nang 550000, Vietnam; 3 Faculty of Medicine, Duy Tan University, Da Nang 550000, Vietnam; 4 Hue Central General Hospital, Hue 52000, Vietnam; 5 Institute of Health Economics and Technology (iHEAT), Hanoi 100000, Vietnam; 6 EA 3279, CEReSS, Research Centre on Health Services and Quality of Life, Aix Marseille University, 27, Boulevard Jean-Moulin, CEDEX 05, 13385 Marseille, France; 7 Bloomberg School of Public Health, Johns Hopkins University, Baltimore, MD 21205, USA; 8 Department of Psychological Medicine, Yong Loo Lin School of Medicine, National University of Singapore, Singapore 119228, Singapore; 9 Institute for Health Innovation and Technology (iHealthtech), National University of Singapore, Singapore 119077, Singapore

**Keywords:** cancer, health utility, interventions, HRQL

## Abstract

Cancer led to nearly 10 million deaths in 2020, as reported by the World Health Organization (WHO). Consequently, both biomedical therapeutics and psychological interventions have been implemented to decrease the burden of this non-communicable disease. However, the research conducted so far has only described some aspects of these interventions, which may increase the health-related quality of life of cancer patients. Therefore, a systematic review is necessary to depict an overall picture of the cancer interventions globally. Then, the impact of these interventions on the preference-based health-related quality of life of cancer patients may be synthesized. The protocol is developed using the Preferred Reporting Items for Systematic Reviews and Meta-Analyses guidelines. The Web of Science database is used to retrieve the literature using four keyword terms: quality of life (QoL), cancer, interventions, and health utility. Then, we draw the global mapping diagram and conduct the meta-analysis for this research. Additionally, longitudinal measurements are used to estimate the changes in the health utility of patients during the interventions. Thus, this systematic review can provide insight into the impact of interventions on increasing the health-related quality of life (HRQL) of cancer patients.

## 1. Introduction

A report from the World Health Organization (WHO) shows that cancer has become the leading cause of human death over the last few years. Specifically, cancer caused nearly 10 million deaths in 2020, which means that the rate is one in every six deaths [1,2]. Cancer has many different types and is one of the leading non-communicable diseases globally. Scientists have discovered more than one hundred different types of cancer, but most of them are mutations that cause cells to become disordered and to proliferate uncontrollably due to different causes such as the environment, living habits, or heredity. Currently, some kinds of cancer can be cured if they are detected and treated early. Some common cancer types, such as breast cancer, cervical cancer, oral cancer, and colorectal cancer, have high cure probabilities when detected early and treated with suitable methods. However, this disease has had many bad effects on human mental and physical health for a long time, which can significantly decrease the health-related quality of life (HRQL) and the finances of patients [3]. In Vietnam, the Ministry of Health reported that there were approximately 182,563 new cancer cases and 122,690 people died because of cancer in 2020 [4]. They estimated that there were 159 people with cancer and 106 people among them died this year [4]. The cancer control program has been applied in Vietnam for more than 20 years, but the results are still very limited.

Additionally, the concepts of quality of life (QoL) and health-related quality of life (HRQL) were mentioned by World Health Organization (WHO) in recent years. There are many aspects of QoL that have been studied, but among the issues of most concern are physical health, physiological function, mental health, social roles and functions, individual’s perception of health, and characteristic symptoms. To evaluate the aspects of QoL, we need to consider both subjective and objective factors, from which we can determine the approach to measurement, selection of measurement tools, measurement design, and the processing, analysis, and interpretation of the results. The effort of making improvements may be vital for patients with certain fatal diseases, such as some types of severe cancer [5]. The definition of a quality-adjusted life year (QALY) is a measure of human health outcomes regarding disease burden and is used to assess the value of medical interventions [6]. Additionally, a quality-adjusted life year measures how much additional time of life of reasonable quality a patient or person may gain due to treatment. The QALY metric is essential in health economics, medical cost calculations, program evaluations, and insurance coverage determinations [6].

These days, some modern cancer interventions and treatments that are developing rapidly can help to eliminate the bad effects and increase the QoL and HRQL of patients with this non-communicable disease [3]. However, the cost of cancer interventions or treatments may be quite expensive, especially for developing countries and low-income patients. Some researchers used health economic analysis to assess the different types of cancer interventions, and made suggestions to clinicians, cancer patients, and policymakers regarding the most suitable methods [7,8]. These cancer interventions can be classified according to different approaches, for example, non-pharmaceutical, pharmacological technique, social contact, psychological therapy, family-based communication, and invasive therapeutics. Some pharmacological techniques and invasive therapeutics have been applied widely in cancer treatment; however, they may have some dramatic side effects on patients, and the cost is another notable problem [9]. Therefore, clinicians often have to combine these methods to increase the effectiveness of treatment for cancer patients. Current palliative care is developing significantly in the world. Palliative care has some differences from hospice care, as it is only focused on increasing the patient’s quality of life when curative treatment is no longer the goal of care [2,10]. This method is a philosophy of care focused on the improvement of the QoL of patients and their family members as a process of coping with the burden of chronic disease, even death [11]. Moreover, cancer interventions may include primary prevention, when people try to eliminate the cancer exposure factors [12], and a screening program, which can help people to detect cancer at the early stages [13]. These types of interventions are now developing rapidly as an essential duty to decrease the incidence and the burden of cancer. Therefore, the research trend may have changed from invasive and curative care in the past to palliative and prevention care in recent years.

Economic assessment is important in different countries because it enables governments to stabilize health financing systems while helping to support expansion in both the size and quality of services in the health sector [14]. Furthermore, there are some differences in the health assessment of some countries due to the variety of different cultures, policy, and the structure of governments [15,16]. Although studies on health economic evaluation are commonly applied in developed countries and mentioned in systematic reviews by some scientists, the extent of application of these studies is still limited, especially in developing countries [16,17,18]. Health utility measurement is a part of health economic assessment [17]. The main aim of health utility measurement is to provide an indicator of the output of a health intervention program. Thus, it can provide general health information for patients and preference-based frameworks for the objective to measure the function of health-related quality of life and create utility scores [19,20,21]. This approach includes four components: a health status classification system, a preference-based scoring function, data collection questionnaires, and coding algorithms for deriving HUI variables from responses to the questionnaires. From that, they can estimate the quality-adjusted life years and the health-related quality of life [8,16,17,22]. Therefore, it can help the therapist understand the psychological and physical status of the patient as well as the patient’s willingness to describe their health status. Then, the researchers can apply this method to find the most effective combined pharmacological and non-pharmaceutical interventions for cancer patients at an early stage or a late stage, especially for vital interventions at the end-of-life stage [5,23,24].

However, the literature conducted so far has discussed several aspects of the impact of cancer interventions, which can change the QoL and the HRQL of patients. A previous systematic review was conducted to assess the health-related quality of life in Asian patients with breast cancer under different treatment methods. In this research, the authors found that patients with comorbidities or who receive chemotherapy are more likely to experience poorer HRQL [25]. In addition, another study involving prostate cancer patients under primary intervention mentioned that the data identified differences in the HRQL on various scales: physical wellbeing, social roles and functions, vitality, and emotional roles [26]. These studies may only deeply evaluate one aspect of the measure of the quality of life of some serious cancers, but they lack the evaluation of the overall relationship between the two concepts above [7,27]. Indeed, some studies showed that actions in the cancer prevention program may be fragmentary, uncoordinated, and unsystematic [28,29]. It can be difficult to decide on the cancer intervention and health utility measurement because of the influence of factors such as economics, insurance policies, and the complex nature of each type of cancer [17]. In particular, the cut-off point for severe, moderate, and mild cancer patients and non-cancer patients is different, which can significantly affect changes in the health utility score of cancer patients during the time of follow-up [30]. Meanwhile, to our knowledge, it is necessary to do a more comprehensive and systematic assessment of cancer intervention and health utility to help cancer patients and doctors to increase treatment outcomes [28,29,30]. With regard to literature mining, this research can exploit data from previous studies, thereby further expanding the assessment of the quality of life of cancer patients under the influence of interventions [31]. Our systematic review can focus on the evaluation and estimation of the trend of cancer interventions used in the world, and describe the change in cancer patients’ QoL and HRQL during the application of these interventions. Our research aims to answer two questions:

What are the global situation and tendencies of cancer intervention programs and health utility measurement applications to develop the quality of life of cancer patients?

How can the impact of these cancer interventions on the health utility of patients be synthesized?

## 2. Materials and Methods

**Figure A1:**
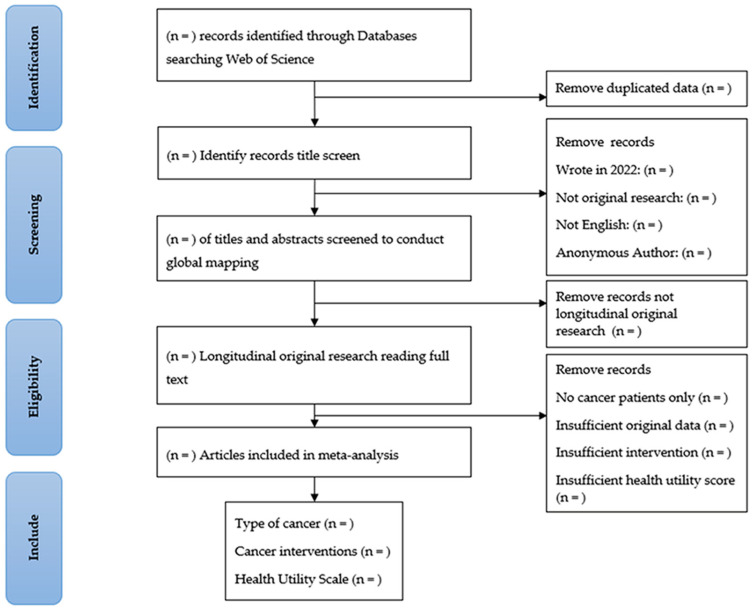
Diagrammatic presentation of the study’s selection process for systematic review.

Firstly, this systematic review followed the Preferred Reporting Items for Systematic Review and Meta-Analysis Protocols (PRISMA-P) framework. Figure A1 presents a full checklist of PRISMA-P [32]. In this figure, we describe the study selection process. Firstly, all records are identified using keyword terms in the Web of Science (WOS) database. Then, we can screen to remove the duplicated articles and apply the inclusion and exclusion criteria to filter the titles, abstracts, and full text of these papers. After that, the included studies are considered the extracted data. At this point, we can conduct the systematic reviews and meta-analysis.

### 2.1. Database

Web of Science (WOS) was chosen for our research because it has more advantages than some other research resources, for example, PubMed, Cochrane, and Scopus [33], including that:

- It can allow a large number of full-text papers, with some that cannot be accessed in other databases.
- It covers scientific publications since 1900.
- It includes high-impact scientific journals from all over the world.

### 2.2. Eligibility Criteria

In this study, we collected the pilot data according to Table A1 until the end of 2021 because the incomplete data from 2022 could not reflect the general research trend and information for the whole year. However, in a later review to be conducted on a specific day in 2023, the study should collect data until the end of 2022.

The following is a description of the literature searching strategy:

- Step 1: We used the combination keyword terms in Table A1 to extract the articles, which mention quality of life and wellbeing in their title, abstract, topic, and keywords.
- Step 2: From the scientific research extracted in the first step, terms related to cancer, cancer intervention, and health utility measurement were used. Then, after filtering on the WOS database, we reviewed it again, with two people independently reading the title and abstract and removing research articles that did not match the inclusion and exclusion criteria outlined below. After that, we continued to read the full text of those studies to carefully filter them one more time using the exclusion criteria to extract the data. Any disagreements between the researchers were resolved by reviewing the text with another senior researcher.

#### 2.2.1. Inclusion Criteria

- The articles were published before 2022.
- They must have full text, full abstract, and full original data.
- They were written and published in English.
- Their content is directly related to cancer, health utility measurement, and cancer intervention impact on the quality of life of cancer patients.
- The study design must be original research to conduct the global mapping and longitudinal research and to conduct the meta-analysis.
- The data in the included articles should describe the change in the health utility score of cancer patients during the time of follow-up. These are described in a table with mean, median, standard deviation, standard error, 95% confidence interval, risk ratio, odds ratio, and interquartile range.

#### 2.2.2. Exclusion Criteria

- Articles published in 2022.
- Articles that are e-papers, only abstracts, opinions, letters, ecological articles, non-human research, advertisements, and conference proceedings.
- Articles written in another language.
- Articles that only include secondary data.
- Articles that do not only focus on cancer patients.
- The data are only described by figures and trends.
- Missing follow-up data.
- Lack of information or information not related to cancer, cancer intervention, and health utility measurement.

### 2.3. Quality of Included Publication Assessment

To conduct the meta-analysis, the longitudinal study design was the only one chosen because we wanted to assess the impact of the interventions on the change of health utility score of cancer patients after a time of follow-up. Then, we chose the Newcastle–Ottawa scale (NOS) and the Jadad scale to evaluate the quality of the included articles [34].

The Newcastle–Ottawa scale was chosen to assess the quality of the non-randomized control trial study [35]. This scale has three main parts: selection, comparison, and outcome or exposure. The selection part has four questions and the maximum points available are four. The comparison has one question with two parts and the maximum points available are two. Finally, the outcome section has three questions and the maximum points available are three. After that, the final scores were divided into three ranks: good quality (3 or 4 points in the selection section, 1 or 2 points in the comparability section, and 2 or 3 points in the outcome/exposure section), fair quality (2 points in the selection section, 1 or 2 points in the comparability section, and 2 or 3 points in the outcome/exposure section), and poor quality (0 or 1 points in the selection section, 0 points in the comparability section, and 0 or 1 points in the outcome/exposure section).

In addition, the Jadad scale, which is an Oxford scale system, can be used to check the quality of the randomized control trial study design [36]. This scale consists of three main parts, namely, randomization, blinding, and an account for all patients. The maximum points available for the randomization and blinding sections are three, and the maximum points available for the last section is one. The included publication is a high-quality study if it has at least three points or above, and any with lower points is of poor quality.

All of the total points and ranks of the included articles are compared and described in the table of results.

### 2.4. Data Management

After reading all of the titles, abstracts, and data from the suitable articles three times and discussing them with the senior researchers, we checked the papers for signs of duplication, missing data, or failure to match the research inclusion criteria.

After that, all full texts of these articles were downloaded and added to the EndNote X9 software version 3.3 Bld 12062. Then, we checked the information in the full text, and if the researchers analyzed the same dataset or found a lack of important information, we discussed the issue with the senior researcher before deciding whether to exclude it or not.

### 2.5. Data Extraction

After reading the full text of all included articles carefully, the data were extracted and added to the Microsoft Excel 2013 version 15.0.4753.1003 software. In this case, the data were extracted using the extraction field of customized data presented in Table 1.

### 2.6. Data Synthesis

The descriptive analysis framework can be applied in downloading and extracting the data steps. At that point, the STATA Statistical Software: Release version 16.0 (StataCorp LLC, College Station, TX, USA) can help to describe the fundamental characteristics of the collected articles, which may include the year of publication, the total of citations before 2022, mean citation percentage per year, the total number of papers of these authors published per year, the total number of times these articles were used in the previous 6 months/5 years, and the mean proportion of use in the previous 6 months/5 years [37].

In this study, the model of the Latent Dirichlet Allocation (LDA) command in STATA was applied. The LDA is a familiar and helpful method for topic classification modeling into a group of similar topics [38]. It can separate the collected scientific research as “discrete distributions over latent topics”; each topic can perform as a “discrete distribution over all the terms”. In the first step, we use the STATA software to divide the abstracts and titles of the articles into individual words. Then, these words were randomly attached to one of the n topics with the same opportunities. After every 50 iterations as a burn-in period, these words were assigned to a new topic with a similar theme. When the LDA process was finished, the file with the assigned topics was exported to Excel. Then, two researchers carefully reviewed the title and abstracts of the most cited research on each topic to name each topic manually. Thereby, the LDA method was able to describe the present trends of the research areas in the improvement of the quality of life of cancer patients by applying different interventions.

Moreover, the indexes, the total number, the rate of publications by each topic, and the development in research interests were provided by ranking these topics on the total number of publications in the past 5 years. Table 1 shows the field for the analysis of each type of data.

The software VOSviewer version 1.6.18 was used to draw the global mapping diagram to describe the trends of all research, cancer interventions, and health utility measurement applications. After that, we assessed the data using the following aspects [39]:

- The difference between cancer interventions and health utility measurement between countries based on World Bank data. We classified countries into low-income countries, lower middle-income countries, upper middle-income countries, and high-income countries [40]. Then, the impact and relationship between these groups of countries was depicted as global trends [16,36].
- The relationships between cancer stage (follow TNM and numerical data scale), pharmaceutical and non-pharmacological cancer interventions, and the health utility measurements were evaluated to illustrate the global situation [9].

The application of multiple fractional polynomial models can better describe the association between the duration of the implemented interventions and the change in health utility score, because the amount of data forecast could be numerous. Therefore, it can be difficult for some basic models such as forest plot and funnel plot to analyze this amount of data, and another powerful method should be applied. Thus, multiple fractional polynomial models may be able to compare and evaluate the change of health utility scores among different feature groups such as type of cancer, stage of cancer, type of country, type of health utility scale, and coordination models. In addition, the linear intervention response relationship is often provided by regression models that may not truly reflect the health utility scores at different times of the follow-up; thus, nonlinear curves could be used to increase the flexibility in estimating the trajectories of health utility score changes over time [41].

## 3. Discussion

Research on cancer, interventions, and health utility has been performed in many countries; however, there are only limited global and comprehensive studies on several types of cancer and interventions. Previous reports have focused on research on the incidence and burdens of cancer on patients’ quality of life, which may not cover the global trends or compare between types of cancers and types of interventions.

This protocol was conducted as the initial step of a systematic review and meta-analysis of cancer burden, cancer interventions, and health utility measurement. The review aimed to explore the difference in the application of these terms between developing countries and developed countries [28]. After that, we were able to describe the main trend of cancer research in the world through global mapping. From the data on the number of publications and citations, some predictions can be made about the importance of research related to the impact of interventions on the quality of life of patients. Therefore, we can evaluate which intervention methods have been and are being focused upon.

In addition, the LDA command can analyze the titles and abstracts to describe the most common topic in this field and classify them into different clusters. In addition, this research can help to consolidate the vital health utility measurement in cancer patients. Based on the extracted data, we can show the total number, frequency, and rank of these scales. There are several scales that have been specifically designed to measure the quality of life of cancer patients, such as FACT and EORTC, and these scales are described and compared carefully in our study. After that, the scores of these scales are analyzed using random effect models, which can give information about their similarities and differences. In a study conducted in 2020, the researchers used FACT-Leu as a health utility scale to assess the quality of life of unmet supportive care patients with acute leukemia in China, and they pointed out that consideration when applying interventions is essential to meet the needs of patients [42]. Thus, we believe that this study can be used to assist clinicians in the future through the analysis of the adequacy of scales, intervention groups, and different types of cancer.

The development of a systematic review involving cancer interventions and health utility measurement has been carried out, but most studies have only been performed for a specific health utility scale or type of cancer. However, this protocol of systematic review and meta-analysis may be necessary to perform a broader study, covering different types of cancers, stages of cancer, and different health utility scales. The findings of this kind of research will provide an extensive picture of research trends in global cancer interventions and their impact. Furthermore, the results of the meta-analysis can highlight the differences between some groups of cancers, interventions, countries, and health utility scales. In the research conducted in 2021, the authors discussed health-related quality of life and psychological problems in order to improve the quality of life for patients [43]. The findings are in line with those of this research that illustrate the focused interventions and their distinct impacts on each specific type of cancer.

The results from this completed article will inform and help to guide healthcare researchers and practitioners in terms of the most appropriate and feasible cancer interventions and health utility measurements, which can enhance the treatment effectiveness and survival outcomes of cancer patients. Finally, this global mapping, systematic review, and meta-analysis reveals evidence on how to provide comprehensive care, which can increase the quality of life of cancer patients while following treatment regimens.

However, this study does have some limitations. Firstly, there is a lack of analysis on the quality of life of caregivers and its impact on cancer patients. A study from 2017 assessed some aspects of this field, but the researchers only conducted this research using one specific group of patients in an Italian University Hospital. This research area is worth investing more effort into in further studies [44]. Another limitation of this research is the absence of some demographic features of patients such as marital status, gender, and educational level. These factors were mentioned in some of the previous research, and we believe that more focus in future research in this field is needed [44,45].

## 4. Conclusions

To sum up, this complete research can synthesize the global trend of impact of cancer interventions research on the change of health utility of patients. Besides, several factors should be considered when conducting research in this field. The future research may help to depict a necessity of providing both pharmaceutical and non-pharmacological interventions to improve the quality of life of cancer patients.

## Figures and Tables

**Table 1:** Data extraction fields.

Data Fields	Information
General information	Authors
	Countries/regions
	Journal
	Study design
Study characteristic	Study duration
	Range of ages/group of ages
Research population	Gender proportion
	Type of cancer
Cancer	Stage of cancer
	Type of intervention
Cancer intervention	Population group using these interventions
	Effect of these interventions on the outcome
	Type of health utility measurements
Health utility measurements	Point of scales
	Changes to these points after applying interventions

## Data Availability

Not applicable.

## Appendix A

**Table A1:** Draft of search strategy to be used in online databases.

Terms	Keywords	Results
Term #1	**Quality of life:** TS = “quality of life” OR TS = “well-being”	572,949
Term #2	**Cancer:** TS= “cancer*” OR TS = “metasta*” OR TS = “Oncolog*” OR TS = “oncogen*” OR TS = “carcinogen*” OR TS = “maglinan*” OR TS = “Tumor*” OR TS = “Tumour*” OR TS = “Astrocytoma” OR TS = “Atypical Teratoid” OR TS = “Blastoma” OR TS = “Carcino*” OR TS = “Cholangiocarcinoma” OR TS = “Chordoma” OR TS = “Craniopharyngioma” OR TS = “Ependymoma” OR TS = “Erythroplasia” OR TS = “Esthesioneuroblastoma” OR TS = “Gestational Trophoblastic Disease” OR TS = “Histiocyto*” OR TS = “Leukemia” OR TS = “Lymphoma” OR TS = “Melanoma*” OR TS = “Mesothelioma” OR TS = “Myelo*” OR TS = “Neoplas*” OR TS = “Neuroblastoma” OR TS = “Neurofibromato*” OR TS = “Osteosarcoma” OR TS = “Paraneoplastic” OR TS = “Pheochromocytoma” OR TS = “Rhabdoid” OR TS = “Retinoblastoma”	5,100,939
Term #3	**Intervention:** TS = “intervention” OR TS = “trial” OR TS = “interventions” OR TS = “trials”	2,739,442
Term #4	**Health Utilities:** TS = “health utility” or TS = “health-utility” or TS = “health utilities” or TS = “health-utilities”	2899
Term #5	#1 AND #2 AND #3 AND #4	122
